# Exploring NS5A Protein of Hepatitis C Virus Through Computational Deep Learning Models and Phylogenetic Analysis for Sequence Variability

**DOI:** 10.3390/diagnostics16213517

**Published:** 2026-10-27

**Authors:** Nabeeha Abid, Obaidullah Qazi, Hania Ahmed, Suman Rafaqat, Muhammad Zubair Yousaf

**Affiliations:** 1 KAM School of Life Sciences, Forman Christian College University, Lahore 54600, Pakistan; nabeeha.abid456@gmail.com (N.A.); haniaahmed1499@gmail.com (H.A.); sumanrafaqat55@gmail.com (S.R.); 2 Department of Microbiology, Abdul Wali Khan University Mardan, Mardan 23200, Pakistan; obaidemail@gmail.com

**Keywords:** evolution, hepatitis C virus, LSTM, NS5A gene, genetic variability, machine learning in virology, phylogenetics

## Abstract

**Background:** Hepatitis C Virus (HCV) remains a global health threat, causing severe liver diseases such as cirrhosis and hepatocellular carcinoma. Protein sequences are essential for exploring genetic variability and improving detection. This study presents a generative LSTM model, AI-VNA, for generating and analyzing NS5A protein sequences to enhance understanding of sequence diversity and improve predictive modeling. **Methods:** The AI-VNA model was trained on curated NS5A protein sequences and evaluated against baseline models including CNN, 1D-CNN, GRU, and Simple RNN. Generated sequences were validated using BLAST (version 2.16.0) to assess biological relevance. Additional analyses included a sequence similarity heatmap, phylogenetic clustering using UPGMA, and statistical comparison of sequence similarities via the Mann–Whitney U test. **Results:** AI-VNA achieved 98.56% accuracy, outperforming other models and demonstrating strong capability in modeling sequence patterns. BLAST validation showed an average query coverage of 82%, an average E-value of 1 × 10^−155^, and 96.68% identity to known NS5A proteins. The sequence similarity heatmap revealed notable variability among the generated sequences. Phylogenetic analysis showed distinct clustering patterns, indicating both conservation and diversity. A box plot comparing similarity scores of predicted and database sequences showed significant variation (*p* = 0.01221; Cliff’s Delta = 0.291, small effect). **Conclusions:** These findings highlight the AI-VNA model’s ability to generate evolutionarily relevant and diverse NS5A protein sequences, supporting its potential for improving HCV sequence analysis and aiding in diagnostic and therapeutic developments.

## 1. Introduction

According to a recent World Health Organization (WHO) survey (2024), approximately 50 million individuals worldwide are chronically infected with the Hepatitis C Virus (HCV), with nearly 1 million new infections occurring annually. The global distribution of HCV genotypes exhibits considerable regional variation. Genotype 1 is the most prevalent, accounting for 46% of all HCV infections. Within this genotype, subtypes 1a and 1b are predominantly found in North America, Europe, and Australia. In Japan, subtype 1b is particularly dominant, comprising 73% of HCV cases. Genotype 3, responsible for 30% of global infections, is the second most common and is primarily concentrated in South Asia. Genotype 4 is largely confined to Africa and the Middle East, while genotypes 5 and 6 are geographically restricted to Southern Africa and Southeast Asia, respectively. Notably, individuals infected with subtype 1b exhibit a higher risk of developing hepatocellular carcinoma (HCC) compared to those infected with subtypes 2a or 2c [1]. This global burden exerts significant pressure on healthcare systems, particularly in resource-constrained countries such as Pakistan. However, the analysis of healthcare data remains a complex challenge, requiring the integration of advanced technologies to enable accurate disease prediction and the formulation of effective treatment strategies. Traditional manual methods of data analysis are often time-consuming, labor-intensive, and susceptible to human error. These limitations have prompted the development of automated systems designed to enhance diagnostic accuracy and streamline clinical decision-making. While such tools offer promising support across various stages of healthcare delivery, ensuring the accuracy and reliability of the underlying data remains a critical prerequisite for their successful implementation [2]. Among the numerous viral pathogens that challenge global health systems, Hepatitis C Virus (HCV) presents unique molecular complexities. The HCV genome comprises approximately 9.6 kilobases of single-stranded RNA, which is translated into a single polyprotein. This polyprotein undergoes proteolytic cleavage by viral proteases and is further processed by host cellular mechanisms to yield a range of functional viral components. These include three structural proteins—core, envelope glycoprotein 1 (E1), and envelope glycoprotein 2 (E2)—which are essential for viral assembly and host cell entry. In addition, seven nonstructural proteins—p7, NS2, NS3, NS4A, NS4B, NS5A, and NS5B—are generated, each playing a critical role in viral replication, protein processing, and the modulation of host immune responses [3]. NS5A, in particular, is a multifunctional phosphoprotein integral to HCV RNA replication, the modulation of host immune responses, and viral assembly. Its high genetic variability across different HCV genotypes contributes to challenges in both diagnosis and treatment, as certain mutations within NS5A are associated with resistance to direct-acting antiviral agents (DAAs). Despite these challenges, NS5A remains a critical target for therapeutic intervention due to its essential role in the viral life cycle [4]. A major obstacle in the development of an effective vaccine against HCV lies in its extensive genetic heterogeneity. The virus is currently classified into 8 major genotypes and over 86 subtypes, exhibiting up to 30% variation in amino acid sequences across genotypes. This considerable genetic variability significantly complicates vaccine design, as it challenges the creation of a universally effective immunogen [5]. To combat HCV infection, direct-acting antiviral agents (DAAs) primarily target the nonstructural proteins NS5A and NS5B, which are essential for viral replication [6]. The significant genetic variability of the NS5A gene poses challenges for the early detection and effective treatment of HCV. Despite this, NS5A remains a critical target for both diagnostic and therapeutic strategies due to its involvement in viral replication, pathogenesis, and modulation of the host immune response. In this context, deep learning—a powerful branch of artificial intelligence—offers transformative capabilities for analyzing complex biological data. These models can uncover intricate patterns within genomic sequences that traditional computational methods often overlook [7]. Applying deep learning techniques to model NS5A sequence variability holds promise for improving diagnostic accuracy and predicting resistance-associated substitutions, thereby informing more effective treatment strategies [8]. In parallel with deep learning approaches, healthcare professionals increasingly utilize advanced data mining techniques to extract meaningful insights from large-scale clinical datasets. Traditional machine learning algorithms such as support vector machines (SVM), decision trees (DT), random forests (RF), neural networks, linear regression (LR), logistic regression, and k-nearest neighbors (KNN) have been widely applied across various medical domains for disease prediction, diagnosis, and treatment optimization [9,10]. Recent implementations of models such as Support Vector Machines (SVM) and Extreme Gradient Boosting (XGBoost) have demonstrated high predictive accuracy, reaching up to 95% [11,12]. LSTMs capture both short- and long-term dependencies through memory cells and gating mechanisms [13]. This approach integrates advanced computational methods with local epidemiological data to address region-specific HCV challenges, facilitating more effective public health interventions and personalized treatment strategies.

## 2. Materials and Methods

### 2.1. Data Collection

For this study, a dataset of 2200 NS5A protein sequences from HCV genotypes 1 through 7 was compiled from the National Center for Biotechnology Information (NCBI, Bethesda, MD, USA) and the Bacterial and Viral Bioinformatics Resource Center (BV-BRC, Los Alamos National Laboratory, Los Alamos, NM, USA). Genotype 8 was excluded due to the scarcity of complete, high-quality NS5A sequences in public databases, which could compromise model reliability and introduce bias. By including all available sequences from each HCV subtype of genotype 1 to 7, the dataset offers a comprehensive and unbiased representation of the virus’s genetic diversity. Extensive filtering was applied to retain high-quality, full-length sequences (453 amino acids) representing the NS5A protein. The preprocessing phase ensured consistent sequence formatting, accuracy, and completeness, followed by the removal of duplicates and sequences with ambiguous protein residues.

### 2.2. Data Pre-Processing

The data preparation workflow utilized TensorFlow (version 2.11.0, Google LLC, Mountain View, CA, USA) and Keras (version 2.11.0, TensorFlow’s high-level API) to load protein sequences, followed by cleaning to remove extraneous whitespace, normalize case to uppercase, and eliminate empty entries. A unique character set was created, and mapping dictionaries were used to encode each character as a numerical index. Sequences were tokenized using Keras’ tokenizer, converting each protein sequence into integer representations. Output labels were one-hot encoded with Keras (see Figure 1). Padding was applied to standardize sequence lengths. To ensure proper model evaluation, 20% of the data was reserved for validation, while 80% was used for training, allowing the model to learn relevant features before validation [14].

### 2.3. AI-VNA Model Architecture

The Biopython library was employed to extract NS5A protein sequences of HCV from FASTA files. An embedding layer was added to the generative model, implemented as a sequential model in TensorFlow Keras [15], which encodes integer-encoded characters into dense vectors. This was followed by an LSTM layer with 128 units to capture sequential dependencies, and a dense layer using the Softmax activation function to provide probabilities for the next character prediction, making the model trainable. The Softmax function not only improves accuracy but also reduces processing time [16]. The LSTM network utilizes four gates—the forget, update, tanh, and output gates—which enable the effective capture of temporal relationships between input and output data by adjusting weights and activation values [17]. LSTM was chosen due to its ability to effectively model long-term dependencies in sequential biological data. Its gating mechanisms help mitigate issues like vanishing gradients, making it more suitable for complex sequence analysis compared to traditional RNNs, GRUs, and 1D-CNNs. The AI-VNA model was implemented using an embedding layer, a GRU layer with 128 units, and a dense output layer with SoftMax activation. The model was trained using the Adam optimizer and categorical cross-entropy loss function over 10 epochs, with a batch size of 32, using an 80/20 training–validation split. In LSTM-based models, the layers are mathematically written as the forget gate determines how much of the past cell state $C_{t}-1$ should be retained:

$$f_{t}=\sigma (w_{f}x_{t}+U_{f}h_{t}-1+bf)$$

where $w_{f}\;$ and $U_{f\;}$ are weight matrices for the forget gate, bf is the bias term, and *σ* is the sigmoid activation function.

The input gate controls how much new information is stored in the cell state; the equation is as follows:

$$i_{t}=\sigma {(W}_{ix^{t}}+u_{i}h_{t}-1+b_{\dot{l}})$$

A candidate state cell is computed as

$$c_{t}=tanh{(W}_{cx^{t}}+u_{c}h_{t}-1+b_{\dot{c}})$$

where $w_{i}{,U}_{i}{,b}_{i}\;$ are parameters for the input gate, $w_{c},U_{c},b_{c}$ are parameters for the candidate cell state, and tanh is the hyperbolic tangent function.

The cell state equation is as follows:

$$c_{t}=f_{t}.c_{t}-1+i_{t}\cdot {\bar{c}}_{b}$$

The output layer determines the new hidden state:

$$\sigma_{t}=\sigma w_{O\varkappa t}+Uoht-1+b_{0}$$

where $w_{0}{,\;U}_{0},\;b_{0}$ are parameters for the output gate, and $h_{t\;}$ is the updated hidden state.

### 2.4. Training and Evaluation

The model was trained using the TensorFlow Keras API for up to 10 epochs with a batch size of 32 to enhance learning efficiency and performance [18]. A sequence generation function was implemented to produce new sequences from a given seed text using the trained generative model. This sequence prediction approach, commonly used during training, helps evaluate the model’s learning progress while mitigating overfitting and accelerating convergence [19]. Tokens generated at each step were appended to the seed text iteratively until a complete sequence was formed, allowing an evaluation of the model’s ability to learn patterns and dependencies. Categorical cross-entropy loss, typically used in classification tasks, was employed here to measure the divergence between predicted and actual distributions in sequence generation. The Adam optimizer was used for weight updates due to its computational efficiency and adaptive learning capabilities [20,21]. The AI-VNA model was trained using Google Colab with access to a free Tesla T4 GPU. The model contains approximately 1.2 million parameters and requires around 2 min per epoch. and All models were trained with identical parameters to ensure result consistency and comparability, as shown in Table 1.

## 3. Evaluation of Generated Sequences

### 3.1. Sequence Validation

BLASTp was employed to compare the generated NS5A protein sequences against the NCBI non-redundant (NR) protein database to verify their topological and biological relevance. Sequence alignment was assessed using query coverage, similarity was measured through percentage identity, and statistical significance was determined by E-value. The results confirmed that the generated sequences were biologically meaningful and closely aligned with known NS5A proteins.

### 3.2. Phylogenetic Analysis

To evaluate the biological relevance and evolutionary consistency of the sequences generated by the deep learning model, a phylogenetic tree was constructed using the Unweighted Pair Group Method with Arithmetic Mean (UPGMA). This dendrogram compares the predicted sequences labeled as Pred # with reference sequences from a curated database labeled as DB # based on sequence similarity, where distance is calculated as 1—similarity.

### 3.3. Sequence Similarity Analysis

To further evaluate the biological relevance of the generated sequences, a heatmap was constructed to visualize the ordered similarity between predicted NS5A protein sequences (*y*-axis) and reference database sequences (*x*-axis). Pairwise sequence similarities were computed using a scoring function derived from sequence alignment. The similarity score $s_{ij}$ between sequences ⅈ and j was calculated as

$$S_{ij}\;=\frac{M}{L}\times 100$$

where M represents the number of matching residues and L is the total length of the alignment. The resulting heatmap provided a comparative assessment of sequence conservation and variability.

### 3.4. Statistical Analysis

To assess the statistical significance of differences in sequence similarity between the predicted NS5A sequences and the reference dataset, the Mann–Whitney U test was employed. As a non-parametric test, it evaluates whether two independent distributions differ significantly without assuming normality. The U statistic was computed as follows:

$$u=n_{1}n_{2}+\frac{n_{1(}n_{1}+1)}{2}-R_{1}$$

where $n_{1}$ and $n_{2}\;$ are the sample sizes of predicted and reference sequences, respectively, and $R_{1}$ indicates the sum of ranks for predicted sequences. Statistical significance was decided by the resulting *p*-value for the observed differences.

Furthermore, Cliff’s Delta (*δ*) was calculated to quantify the effect size, i.e., to quantify the difference between both distributions:

$$\delta =\frac{\sum (X>Y)-\sum (X<Y)}{n_{1}n_{2}}$$

where X and Y are the predicted and reference sequence similarity scores, respectively.

## 4. Result

### 4.1. AI-VNA LSTM-Based Model

The AI-VNA generative model demonstrated strong learning capability, achieving a peak training accuracy of 98.56%. As illustrated in Figure 2, both training and validation accuracies exhibited consistent upward trends throughout the training process. The training accuracy began at 0.982 and steadily approached its maximum value, reflecting effective convergence and model stability. Correspondingly, the validation accuracy increased early during training, reaching 0.979 at epoch 2, 0.983 at epoch 4, and peaking at 0.984 by epoch 6. Although a slight drop to 0.981 was observed at epoch 8, the model maintained high performance, indicative of robust generalization. Notably, validation accuracy briefly dipped during epoch 7 but recovered at epoch 9, suggesting the model’s resilience to overfitting and its capacity to generalize across unseen data. These results underscore the model’s stability and proficiency in learning complex sequence patterns from the training dataset without significant degradation in validation performance. As a generative model, AI-VNA not only achieves high predictive accuracy but also demonstrates the ability to generate biologically plausible protein sequences. This capability is particularly advantageous for genomic tasks, such as de novo protein sequence generation, where modeling long-range dependencies is essential. Figure 3 highlights the model’s ability to reconstruct and simulate realistic NS5A protein sequences with high fidelity.

This strong performance highlights the model’s robustness in capturing the underlying biological characteristics of the NS5A protein. The AI-VNA model successfully generated sequences with high similarity to known NS5A proteins, demonstrating its capacity to preserve biological relevance in generative outputs. When compared to other sequence modeling approaches, the LSTM-based architecture showed superior performance in both efficiency and accuracy. Specifically, the LSTM model achieved a root mean square error (RMSE) of 68,417 over 15 epochs, outperforming the GRU model, which required 400 epochs and yielded a higher RMSE of 90,173 [18]. This comparison further supports the effectiveness of the LSTM-based generative framework in sequence generation tasks within biological domains.

### 4.2. Sequence Generation of NS5A Protein

Multiple deep learning models were evaluated based on their accuracy and ability to generate biologically relevant NS5A protein sequences. The proposed AI-VNA model achieved the highest accuracy of 98.56%, generating sequences of 453 amino acids—matching the full-length reference NS5A protein—and thus producing outputs that were both structurally consistent and biologically meaningful. In contrast, the 1D-CNN model achieved an accuracy of 86.67%, generating sequences of 402 amino acids. While these sequences approached the correct length, they lacked the reliability and precision demonstrated by the AI-VNA model. The GRU model exhibited a significant drop in performance, with an accuracy of only 42.13%. The sequences it generated frequently contained unnatural repetitions of motifs such as “AAAA” and “GGGG”, indicating a failure to capture the natural diversity present in authentic NS5A sequences. These outputs were biologically irrelevant and structurally inconsistent. Similarly, the simple RNN model achieved an accuracy of 56.00%, producing sequences of 480 amino acids. Although the RNN-generated sequences showed some variation, they were prone to inconsistencies and repetitive patterns, and failed to match the robustness and biological plausibility exhibited by the AI-VNA and 1D-CNN models.

### 4.3. BLAST Analysis of Generated Sequences

All generated NS5A protein sequences were evaluated using BLASTp (Basic Local Alignment Search Tool for proteins) to assess their biological relevance, as summarized in Table 2. A total of 100 sequences were analyzed, each compared against known NS5A protein entries in the NCBI non-redundant database. The analysis revealed an average query coverage of 82%, indicating that most generated sequences aligned well with known NS5A proteins. Importantly, the sequences exhibited an average percentage identity of 96.2%, suggesting that nearly all amino acid positions matched their reference counterparts. Furthermore, the sequences yielded an exceptionally low average E-value of 1 × 10^−140^. Since lower E-values indicate higher statistical significance, this strongly supports the high confidence match between generated sequences and authentic NS5A proteins. Overall, these results reinforce the biological relevance and fidelity of the sequences generated by the AI-VNA model.

### 4.4. Comparison with 1D-CNN, GRU and Simple RNN Models

The comparison between the LSTM based AI-VNA model and the other obtained models in terms of the modeling capability, accuracy and performance of the model, generative quality, and computational efficiency was performed. In comparison to 1D CNN models for generative tasks (such as sequence generation), LSTM not only achieves a higher success rate but also learns significantly faster than traditional recurrent networks [17]. Furthermore, deep architectures have been shown to effectively handle complex tasks involving long-term dependencies. Unlike previous recurrent network algorithms that suffer from the vanishing gradient problem, as they propagate the error signal through many layers, the long-term dependency is handled well through advanced models [22]. The 1D-CNN model achieved an accuracy of 86.67%, demonstrating its effectiveness and producing statistically significant results in the context of sequence generation. This model starts out with an embedding layer that receives input as characters and converts them into dense 50-dimensional vectors, which go into a Conv1D layer with 128 filters and a kernel size of 3 for capturing local patterns in the sequences.

The model includes a GlobalMaxPooling1D layer to down-sample the feature map, a Dense layer with 128 neurons for further feature extraction, and a final output layer (Dense) where the probabilities (or evidence) for each of the character classes are provided using a Softmax activation function implemented via Keras (version 2.11.0, TensorFlow API, Google LLC, Mountain View, CA, USA) to enable the generation of sequences. Now, we need to compile the model using the Adam optimizer and set the categorical cross-entropy loss, the number of epochs, batch size, and validation split as follows. GRU, despite being less complex and yielding similar results (shown in Figure 3), was somewhat outperformed on longer-term dependencies, implying long-term memory with an accuracy of 42.13%. Conversely, ConV1d effectively identifies local patterns and is computationally efficient, but struggles to model long-range dependencies, making it less precise for complex sequences. Vanilla RNN with an accuracy of 56.00% shows a simple approach that does not require many computational resources, but is limited by the vanishing gradient problem and tends to miss long-term dependencies, leading to the least accurate results among these models. We monitor training and validation accuracy, deriving training accuracy from the model predictions on training data and pulling validation accuracy from the training history. AI-VNA is a novel and reliable algorithm for modeling protein sequence variability with high specificity and accuracy. The AI-VNA generative model performed much better than other models analyzed in this research, and achieved a high training accuracy of 98.56% for learning the patterns of NS5A protein sequences.

### 4.5. Evidence of Sequence Diversity in Predicted NS5A Sequences

To achieve this, we generated the heatmap of sequence similarity between 10 sequences from the database and 10 sequences generated by the AIVNA model, as presented in Figure 4. The results show that many predicted sequences closely match those from a database, having values for similarity over 0.9 in some cases (ex. database 2 vs. predicted 1, database 5 vs. predicted 5, database 6 vs. predicted 6). This exemplifies the model’s capability to produce biologically relevant sequences resembling actual NS5A sequences. Few of the predicted sequences have moderate to lower similarity scores (e.g., database 6 vs. predicted 3, database 7 vs. predicted 6 with scores of 0.50, 0.55, respectively), reflecting variations that probably attune to new structural patterns or a certain divergence among sequences. Clustered similarity patterns additionally indicate that certain predicted sequences include conserved characteristics across several database sequences. The heatmap illustrates the advantage of models like AIVNA, which generates sequences with higher and rather homogeneous similarity scores when compared to traditional models like 1D CNN, GRU, and Simple RNN.

### 4.6. Phylogenetic Analysis of Generated Sequences

A phylogenetic tree was constructed using the UPGMA (Unweighted Pair Group Method with Arithmetic Mean) clustering algorithm to compare the predicted sequences generated by the deep learning model with reference sequences obtained from a curated database. The dendrogram shown in Figure 5 illustrates the evolutionary relationships based on sequence similarity, providing insights into the fidelity and biological relevance of the generative model. Several predicted sequences demonstrated strong phylogenetic proximity to their respective database counterparts. For instance, Pred_3 exhibited near-identical similarity to DB_3, indicating highly accurate sequence replication by the model. Similarly, Pred_1, Pred_2, and Pred_5 clustered closely with DB_1, DB_2, and DB_5, respectively. Notably, Pred_6 initially grouped with Pred_2 before merging with DB_2, suggesting internal consistency among the predicted sequences and reinforcing their alignment with known biological patterns. In contrast, some sequences displayed moderate divergence. Pred_7, Pred_9, and Pred_10 formed separate clusters before eventually merging with DB_7, DB_9, and DB_10, respectively, implying potential novelty or a representation of less common variants. These clustering patterns highlight the model’s ability to capture and replicate biologically meaningful sequence motifs while also demonstrating the capacity to generate sequences that explore novel evolutionary space. The close association of most predicted sequences with established references confirms the model’s competency in learning structural and functional features of the viral genome. Meanwhile, the more divergent sequences may reflect variant formation or mutational drift and merit further experimental validation to determine their biological plausibility and relevance.

### 4.7. Assessing Sequence Similarity and Variability Through a Mann–Whitney U Test

To evaluate the similarity between the predicted peptides and the sequences in the databases, a Mann−Whitney U test was conducted. Indeed, given the initial distributions in the database and predicted sequences that we saw in the run, after performing the test we found that the *p* value came out as 0.01221, indicating a noticeable difference between the two distributions. Nonetheless, the effect size measured by Cliff’s delta was 0.291, indicating a small but statistically notable difference between the generated and known NS5A sequences. This quantifies the degree of variability captured by the AI-VNA model in comparison to native protein sequences. The predicted sequences also have a higher median similarity score with a narrower distribution, as shown in the box plot, suggesting that they yield more consistent patterns (Figure 6). In comparison, the sequences in the database are more varied, reflecting naturally occurring sequence divergence. These findings indicate that the predicted sequences were confirmed to be on-target with the known sequences, though slightly differing as well, providing the basis for their potential in future therapeutic development. The diversity detected in predicted sequences could contribute to adaptability and may indicate possible evolutionary variations, making them interesting for further studies in HCV.

## 5. Discussion

One of the key challenges in bioinformatics today lies in the generative modeling of proteins to support advancements in synthetic biology, medicine, and material science [23]. The integration of computational approaches is transforming the landscape of protein design, offering innovative solutions for challenges such as drug resistance prediction and the development of novel therapeutics. Generative models not only simulate the natural diversity of proteins but also hold the potential to drive breakthroughs in precision medicine by offering insights into protein structure, function, and interactions. The model’s capacity to learn complex biological patterns enables the exploration of sequence space far beyond what is accessible through traditional experimental methods. Moreover, machine learning techniques have already demonstrated impressive results in virology. For instance, ref. [24] reported that their SVM model achieved an average accuracy above 80% in distinguishing between human papillomaviruses (HPV) and hepatitis C virus (HCV). This performance surpassed previous SVM-based models employing different feature representation schemes, indicating the effectiveness of tailored machine learning frameworks in viral tasks [15]. In light of these developments, the promising results from our generative approach, particularly the AI-VNA model, reinforce the potential of deep learning in accurately simulating HCV NS5A protein sequences.

This progress is vital for addressing real-world challenges such as overcoming drug resistance, designing more effective vaccines, and enhancing the diagnostic capacity in clinical settings. In this study, we developed and evaluated AI-VNA, a novel LSTM-based generative model specifically tailored for protein sequence generation and analysis. To contextualize its performance, we compared the AI-VNA model against three other deep learning architectures: 1D-CNN, GRU, and Simple RNN. While transformer-based models have shown promise in various sequence analysis tasks, their application typically requires substantially larger datasets and computational resources. Given the scope and dataset size of this study, AI-VNA was selected as a balanced approach offering strong performance with interpretability and feasible computational demands. This study focuses on evaluating AI-VNA against commonly used deep learning architectures well suited for protein sequence modeling within our data constraints. Each architecture presents unique strengths and limitations in the realm of protein sequence modeling. The AI-VNA LSTM model achieved a high accuracy of 98.56% and effectively captured long-range dependencies in NS5A sequences; it is computationally more demanding than simpler models such as GRU or CNN due to its complex gating mechanisms. The 1D-CNN model, known for its ability to learn local spatial features through convolutional filters, achieved a training accuracy of 86.67%. While it demonstrated moderate effectiveness in recognizing short-range sequence motifs, its lack of recurrence mechanisms hinders its capacity to model the long-range dependencies and temporal dynamics intrinsic to protein sequences. Recurrent models such as LSTM and GRU, on the other hand, are inherently designed to capture temporal relationships by maintaining contextual information across time steps. These architectures are better suited for generative tasks, as they can learn the global structure of sequences and preserve the order and dependencies between amino acid residues. Notably, the LSTM-based AI-VNA model outperformed all other architectures in both accuracy and sequence quality, producing biologically relevant and structurally sound NS5A protein sequences. The GRU model, although structurally simpler and computationally more efficient than LSTM, exhibited a substantial performance decline. It generated sequences with frequent repetitive motifs and failed to maintain biological plausibility. Similarly, the Simple RNN model showed limited learning capabilities due to its susceptibility to vanishing gradients and inability to handle long-term dependencies, resulting in suboptimal sequence generation. Deep learning architectures like LSTM, GRU, and CNNs have been extensively utilized in a variety of biological sequence classification and generation tasks, offering capabilities that surpass traditional computational methods. Their ability to uncover complex patterns and correlations in biological data enables predictive modeling that is not easily achievable through manual analysis or conventional statistical techniques [25]. Previous research has demonstrated the successful application of CNN and LSTM models in diverse biomedical domains, such as dengue case prediction [26,27].

While Gated Recurrent Unit (GRU) models are often considered a simplified and computationally efficient alternative to LSTMs, incorporating a gating mechanism to mitigate vanishing gradient problems, their theoretical strengths did not translate effectively in this study. Despite their structural efficiency, the GRU model in our analysis achieved a training accuracy of only 42.13%, indicating its limited ability to learn from complex biological data such as protein sequences. Similarly, the convolutional neural network (CNN) model, although capable of extracting local features through convolution filters, lacks the recurrent memory required to capture long-range dependencies. This imposes architectural constraints on its ability to model complex sequence relationships. In this study, the CNN model achieved a relatively better training accuracy of 86.67%, but still underperformed in generating reliable, biologically plausible protein sequences compared to LSTM-based models.

The Simple RNN model, which retains only the last hidden state and uses a straightforward update mechanism, achieved a modest training accuracy of 56.00%. However, due to its susceptibility to vanishing or exploding gradients, it failed to retain long-term sequence dependencies. This limitation is particularly detrimental for protein modeling tasks, where sequences can span hundreds of residues with intricate patterns and dependencies. As a result, both GRU and Simple RNN architectures were unable to effectively model the structural complexity and diversity of NS5A protein sequences, yielding outputs of limited biological relevance [2]. In contrast, the AI-VNA model based on the LSTM architecture emerged as the most effective generative framework in this study. LSTM’s gating mechanisms, specifically designed to address the vanishing gradient problem, allow for the retention and learning of long-term dependencies—an essential feature when modeling biologically complex sequences such as NS5A. This enabled AI-VNA to achieve a training accuracy of 98.56%, significantly outperforming other architectures. Despite its superior performance, LSTM’s higher computational cost can limit its scalability and accessibility, especially in resource-constrained environments. Future studies could explore more efficient architectures like transformers or optimized recurrent units to balance accuracy with computational efficiency. Similarly, the study in [2] reported that LSTM model outperformed other models in terms of predictive accuracy and sequence generation quality.

These findings lay the groundwork for future studies exploring LSTM-driven sequence generation in other viral proteins, potentially contributing to vaccine development, drug target identification, and the understanding of viral evolution through synthetic biology approaches. Sequence similarity between the AI-VNA-generated sequences and known NS5A proteins from established databases was visualized using a heatmap, which revealed patterns consistent with genetic variability inherent in the HCV NS5A gene. Although minor deviations were observed between the generated and native sequences, these differences align with natural mutations reported in the literature, suggesting that the model captures biologically relevant variability. Thus, the AI-VNA model not only learns to replicate known sequences but also simulates evolutionary features intrinsic to viral adaptation [28]. A phylogenetic tree was constructed using the UPGMA algorithm to evaluate the similarity between the predicted sequences and known database sequences. The clustering, based on pairwise sequence distances (1—similarity), demonstrated that several predicted sequences grouped closely with reference sequences, indicating high sequence fidelity. Notably, clusters such as Pred_3 and Pred_10 with DB_3, and Pred_2 and Pred_6 with DB_2, suggest that the generative model preserved conserved evolutionary features. These findings align with previous studies highlighting the utility of phylogenetic analysis in validating computationally generated biological sequences [29]. These variations are particularly significant in the context of HCV treatment, where NS5A inhibitors are designed to target conserved regions of the protein. Mutations within these regions can disrupt drug binding, thereby inducing resistance [30]. In our analysis, the Mann–Whitney U test (*p* = 0.01221) revealed a statistically significant difference between the similarity distributions of the predicted sequences and the known NS5A sequences in the database. Although the reported effect size (Cliff’s delta = 0.291) indicates a small magnitude of difference between generated and known NS5A sequences, even subtle variations in viral protein sequences can have significant biological consequences. In the context of HCV, minor sequence changes within conserved regions may influence drug binding affinity, viral replication efficiency, and immune evasion. Therefore, while the statistical effect is modest, the biological impact could be substantial, especially given the complex interplay between sequence variation, host–virus interactions, and the critical importance of sequence-specific analysis in understanding NS5A function, particularly given that NS5A phosphorylation states are directly linked to viral replication fidelity [31]. Overall, these results are consistent with a nascent but increasingly substantial body of work highlighting the key role of deep learning in not only describing virality within species genetic variation, but also viral evolutionary dynamics, host–pathogen interactions, and mechanisms of drug resistance.

This work facilitates deeper insights into the inherent variability of the NS5A protein, which is crucial for identifying conserved regions that can be targeted in the development of specific antiviral drugs or vaccines. By simulating diverse NS5A protein sequences, the AI-VNA model also offers a powerful approach to understanding the molecular basis of drug resistance, thereby enabling the design of more effective, precision-targeted therapies against HCV. Notably, patients with chronic HCV infection often respond to antiviral therapy with an initial sharp decline in viral load, followed by a gradual reduction until the virus becomes undetectable—a therapeutic pattern well documented in clinical studies [32]. These capabilities highlight the transformative potential of deep generative models in advancing both fundamental biological research and translational biomedical applications.

## 6. Conclusions

The AI-VNA model based on the LSTM architecture outperformed the 1D-CNN, GRU, and Simple RNN models and was capable of generating biologically meaningful protein sequences. This highlights the importance of architecture selection as not all architectures handle these challenges of protein sequence modeling adequately. While the observed effect size was modest, the biological relevance of the generated sequences supports the model’s utility. Limitations such as computational demands and the need for broader validation should be considered in future work. LSTM-based models have a lot of potential and the results of this particular study improve the understanding of viral proteins (in this case NS5A) while also contributing to the overall discourse surrounding protein engineering, drug discovery, and disease research within the realm of computational biology.

## Figures and Tables

**Figure 1:**
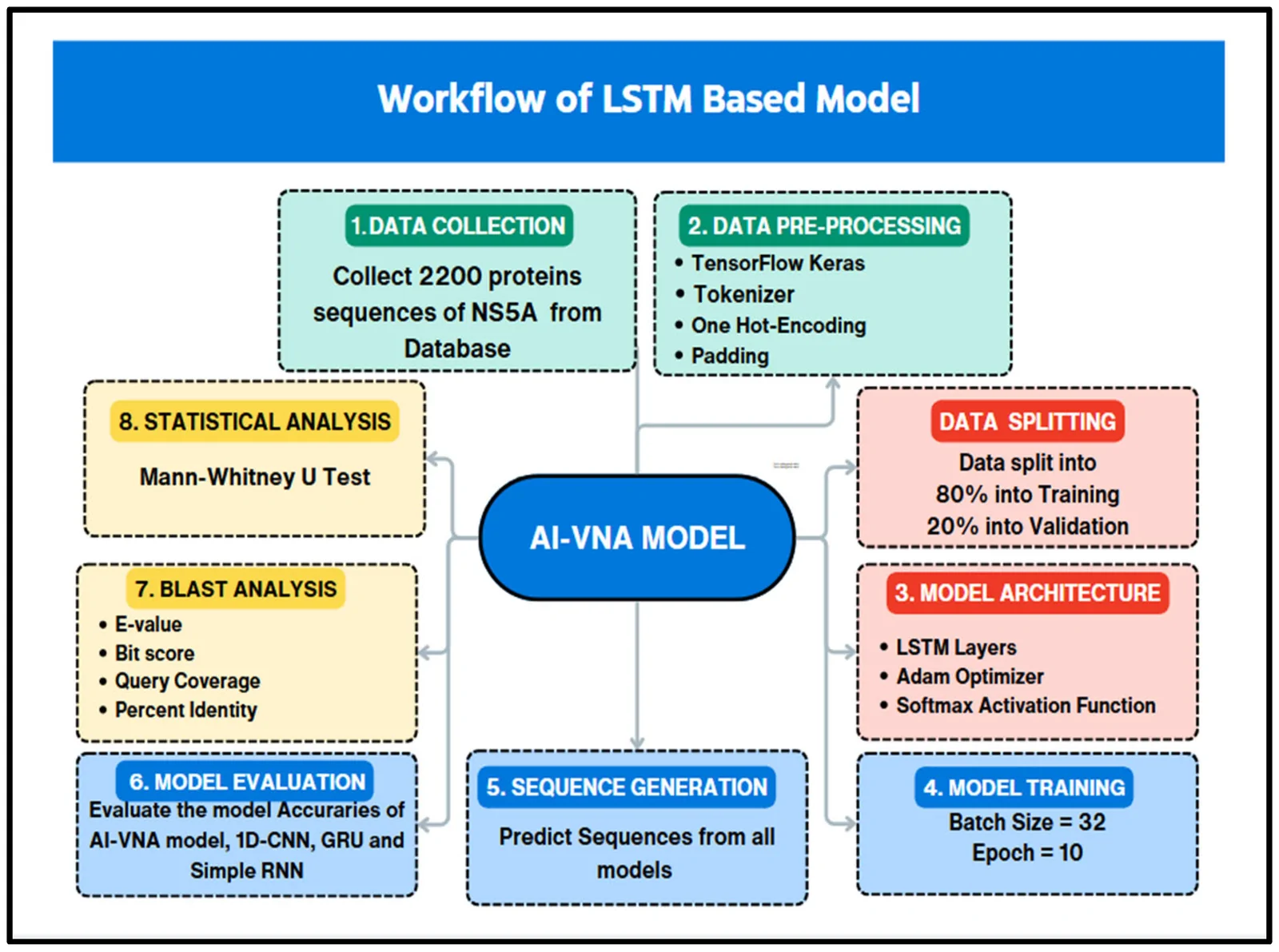
Schematic diagram illustrating the workflow of the AI-VNA model: In the first stage we obtain 2200 NS5A protein sequences, followed by a preprocessing stage using TensorFlow and Keras in which we one-hot encode the sequences and pad them to the same length. This step advances the input data to a format that can be used for deep learning. These processed sequences are then used to train a model using TensorFlow Keras framework. The model is trained on 32 batch sizes and for 10 epochs to capture sequence patterns effectively.

**Figure 2:**
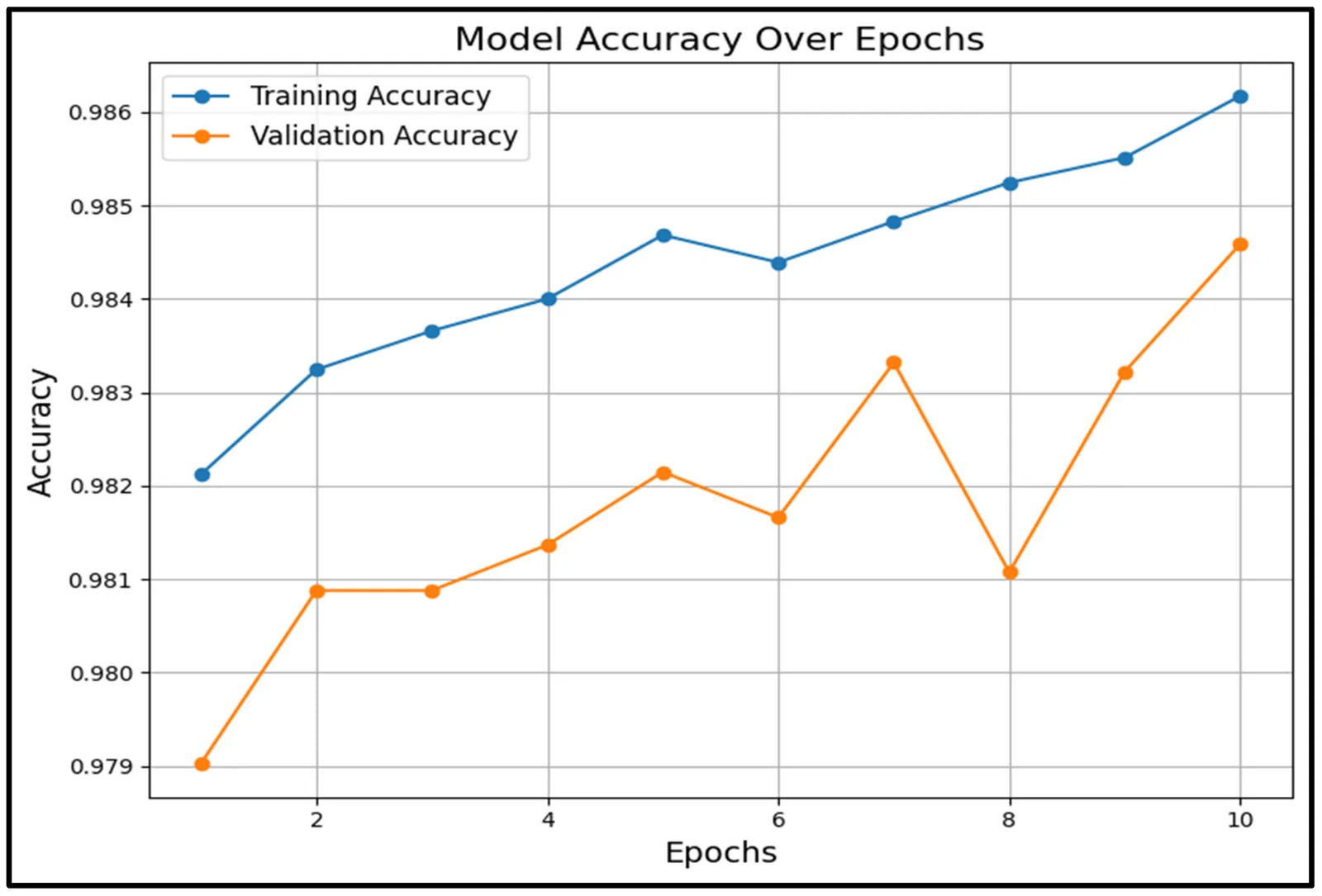
Performance evaluation of the AI-VNA LSTM model. This figure displays the accuracy curve of the AI-VNA LSTM model during training and evaluation on the NS5A protein sequence dataset. The training graph illustrates the model’s performance across 10 epochs, highlighting the progression of accuracy over time.

**Figure 3:**
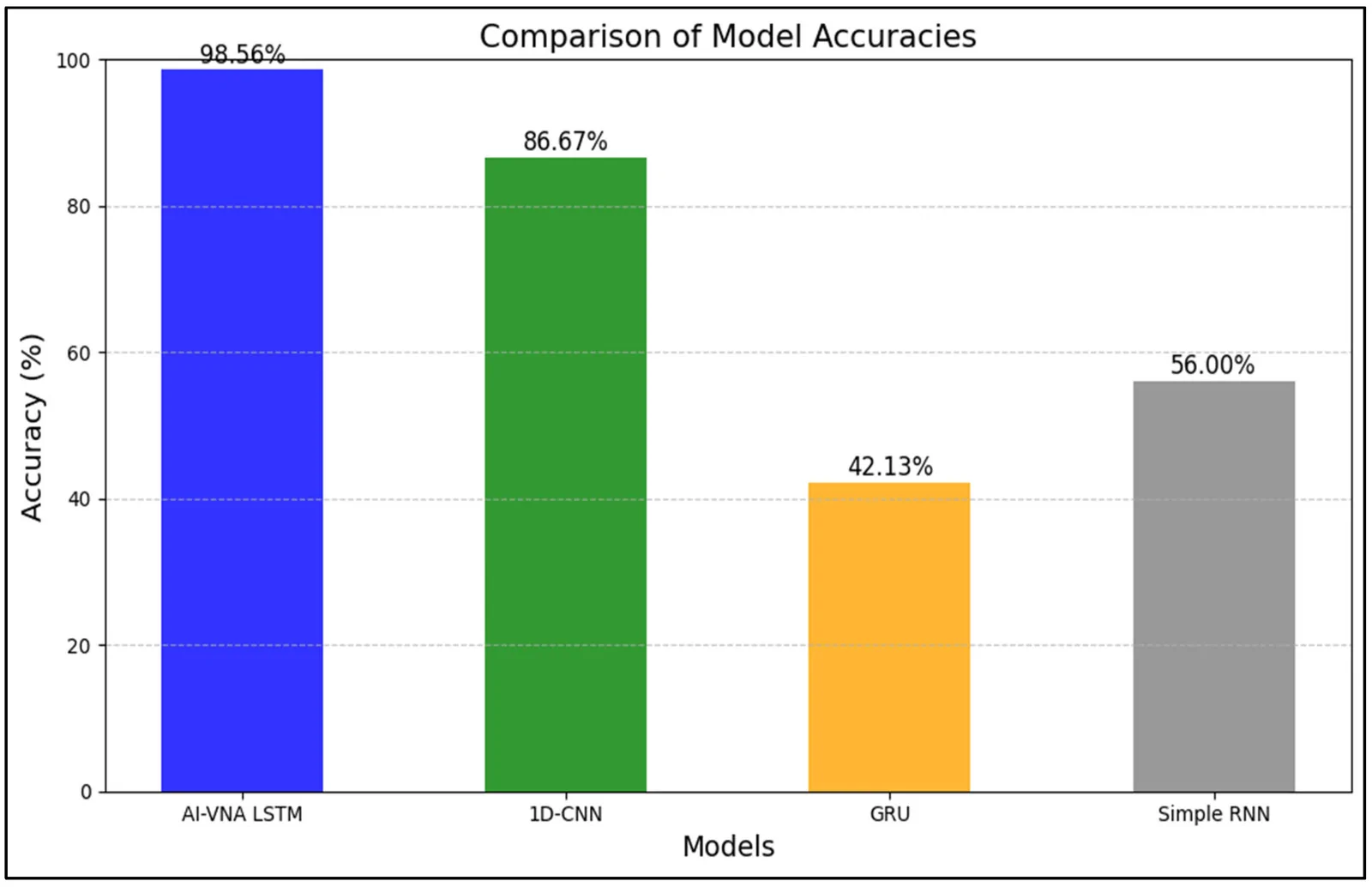
Accuracy comparison of AI-VNA, 1D-CNN, GRU, and Simple RNN models. This figure presents a bar graph comparing the accuracy of four deep learning models—AI-VNA, 1D-CNN, GRU, and Simple RNN—on the NS5A protein sequence dataset. The graph visually contrasts the performance of each model, illustrating how each architecture performs in terms of accuracy after being trained with a batch size of 32 for 10 epochs.

**Figure 4:**
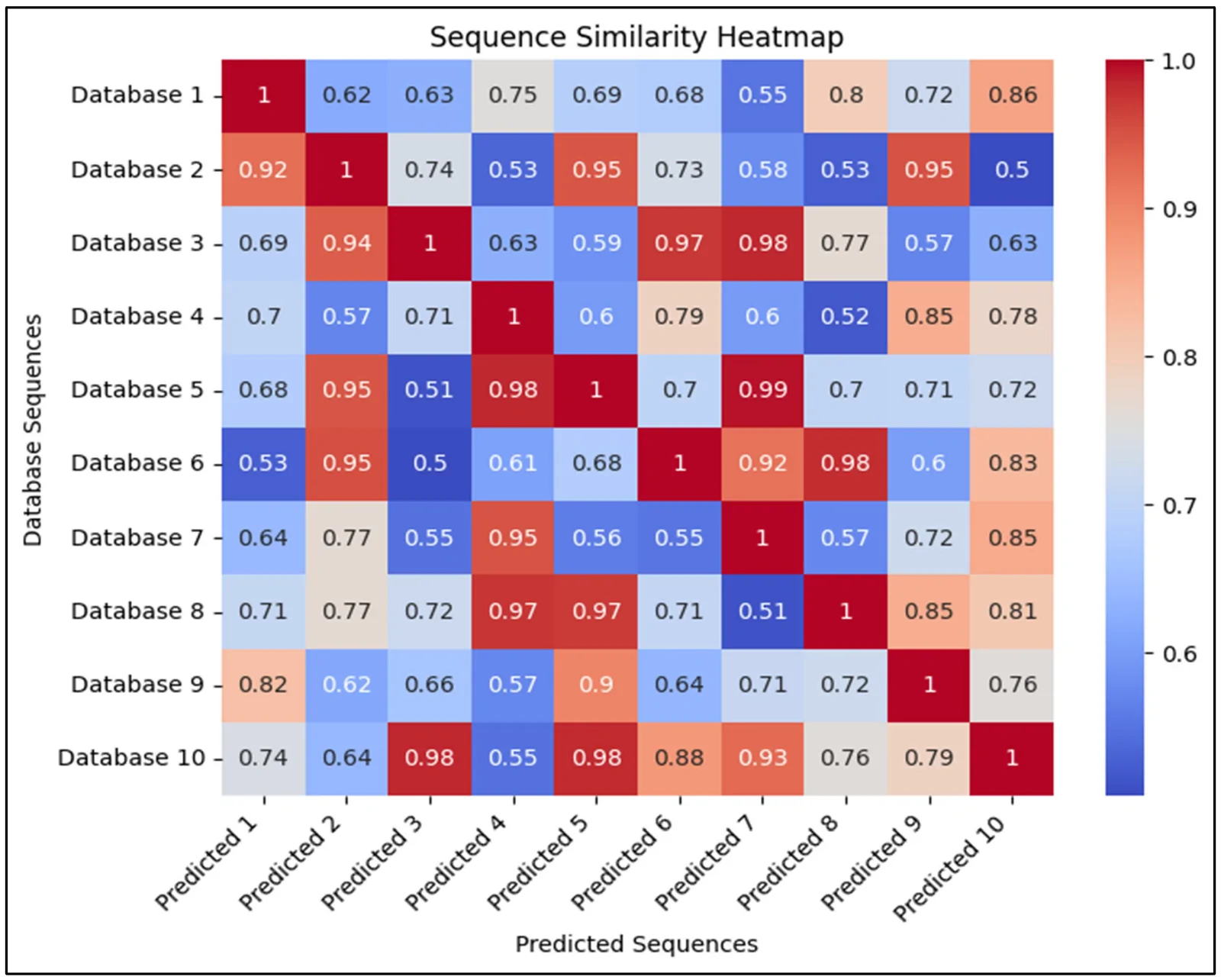
Sequence similarity analysis of database and predicted sequences. Heatmap visualization of two-dimensional comparison method between 10 database sequences and 10 predicted sequences for AIVNA model. Dark red means high similarity, and dark blue low similarity. Predicted sequences are on the x-axis and database sequences on the y-axis. Each sample pair is assigned a score, reflecting how well the two sequences in question match, from zero as no match to one as a perfect match.

**Figure 5:**
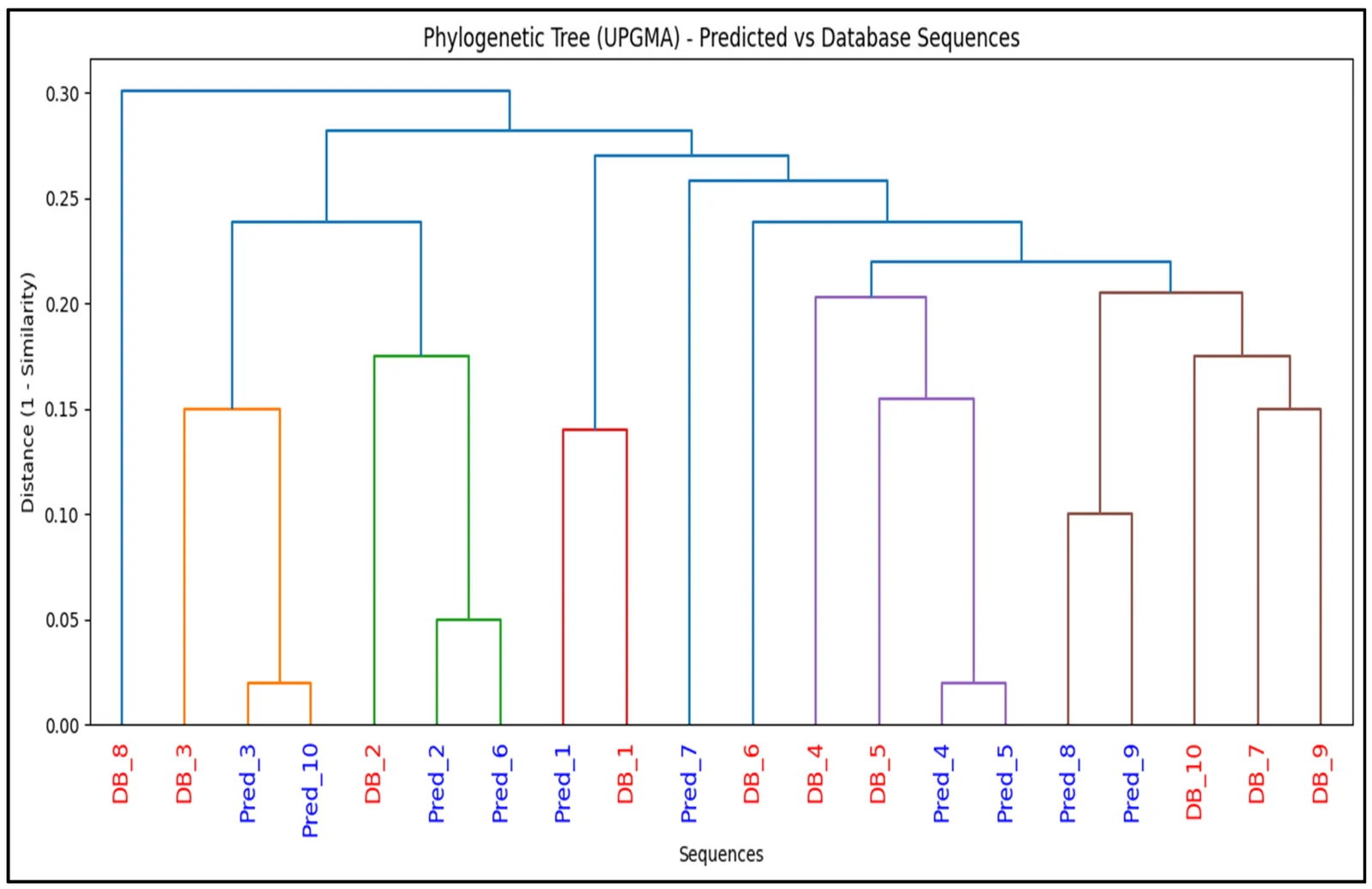
Evolutionary relationships of NS5A protein sequences based on phylogenetic analysis. The dendrogram represents a phylogenetic tree constructed using the UPGMA (unweighted pair group method with arithmetic mean) algorithm, comparing predicted sequences (Pred_#) and reference database sequences (DB_#) based on sequence similarity. The y-axis indicates the distance (1—similarity).

**Figure 6:**
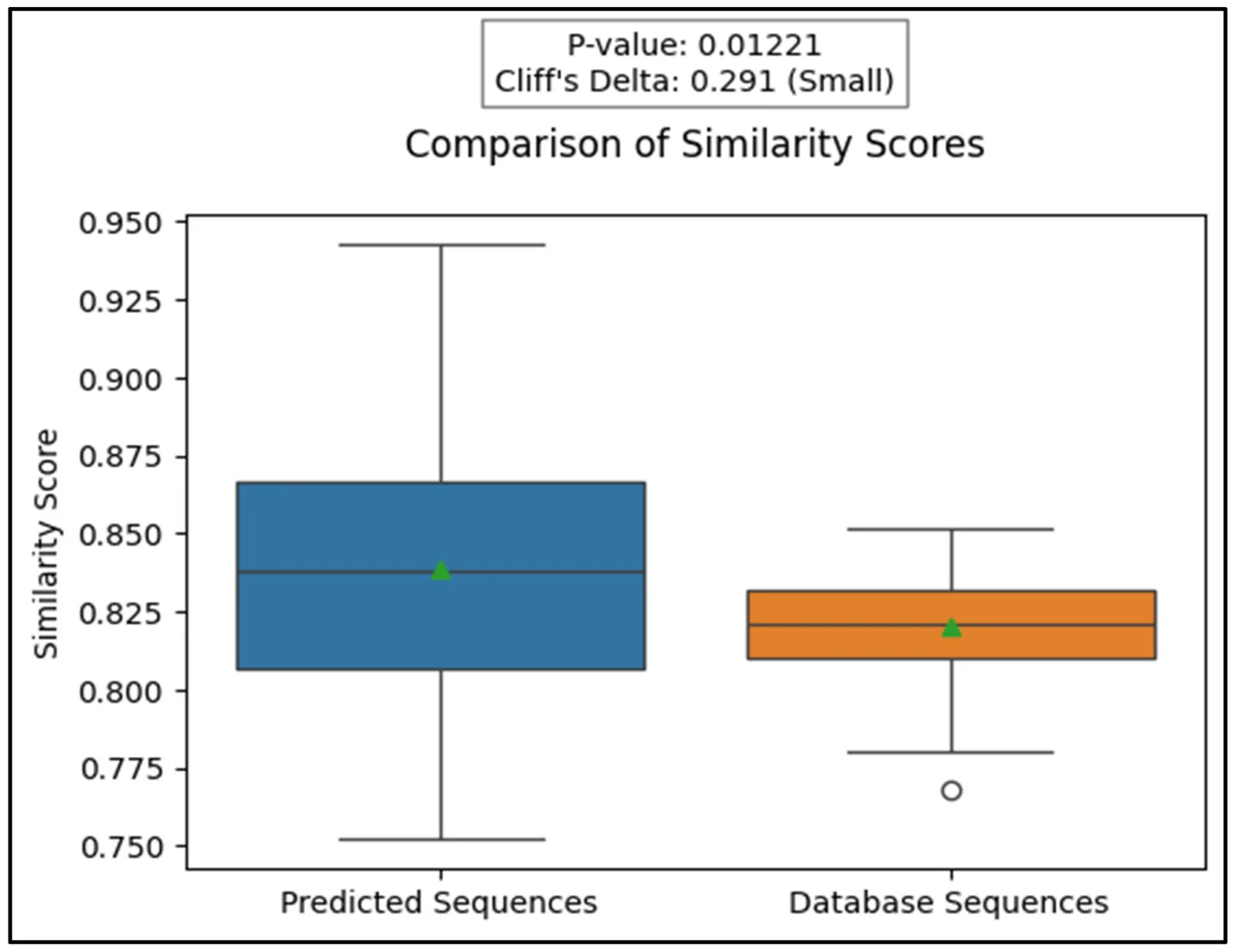
Comparison of similarity scores between predicted and database sequences. In the box plot, the distribution of the similarity scores of predicted and database sequences is shown. Predicted sequences are shown in blue boxes while database ones are represented with orange boxes. The green triangle (▲) represents the average similarity score per group. The whiskers illustrate the spread of the values, whereas the box shows the interquartile range (IQR) and the horizontal line indicates the median.

**Table 1:** Performance comparison of AI-VNA, 1D-CNN, GRU, and Simple RNN models. The table compares the performance of four deep learning models with respect to layers, batch size, optimizer, epoch and accuracy.

Sr. No	Parameters	AI-VNA	1D-CNN	GRU	Simple RNN
1	Layers	Embedding, LSTM, Dense	Embedding, Conv1D, Dense	Embedding, GRU, Dense	Embedding, Simple RNN, Dense
2	Optimizer	Adam	Adam	Adam	Adam
3	Epoch	10	10	10	10
4	Batch Size	32	32	32	32
5	Units Per Layer	128	128	128	128
6	Activation Function	Softmax	ReLU (Conv1D)/ Softmax/Dense)	Softmax	Softmax
7.	Loss Function	Categorical Cross-entropy	Categorical Cross-entropy	Categorical Cross-entropy	Categorical Cross-entropy
8.	Training Split	80%	80%	80%	80%
9.	Validation Split	20%	20%	20%	20%
10.	Recurrent Layers	LSTM	ConV1D	GRU	Simple RNN

**Table 2:** BLAST analysis results of generated NS5A protein sequences. This table summarizes the BLAST analysis metrics for the 100 generated NS5A protein sequences, including query coverage, E-value, and percentage identity, to assess their similarity to known NS5A sequences.

Metric	Results	Explanation
Total Sequences Generated	100	The number of NS5A protein sequences analyzed.
Average Query Coverage	82%	On average, 82% of each generated sequence aligned with known NS5A sequences, indicating partial but significant similarity.
Average E-Value	1 × 10^−140^	A very low E-value, signifying a highly significant match between generated and real NS5A sequences.
Average Percentage Identity	96.2%	Indicates that, on average, 96.2% of amino acids in the generated sequences were identical to those in known NS5A proteins, demonstrating high similarity.

## Data Availability

All the codes used in this study can be found in the GitHub (version 3.14.7) repository https://github.com/NabeehaAbid456/AIVNA (accessed on 25 January 2025).
